# Identification of *Salmonella* Serogroups and Distinction Between Typhoidal and Non-Typhoidal *Salmonella* Based on ATR-FTIR Spectroscopy

**DOI:** 10.3390/microorganisms12112318

**Published:** 2024-11-14

**Authors:** Maira Napoleoni, Stefano Ceschia, Elisa Mitri, Elisa Eleonora Beneitez, Valentina Silenzi, Monica Staffolani, Elena Rocchegiani, Giuliana Blasi, Elisa Gurian

**Affiliations:** 1 Centro di Riferimento Regionale Patogeni Enterici Marche, Istituto Zooprofilattico Sperimentale dell’Umbria e delle Marche “Togo Rosati”, Via Maestri del Lavoro, 7, 62029 Tolentino, Macerata, Italy; e.beneitez@izsum.it (E.E.B.); v.silenzi@izsum.it (V.S.); m.staffolani@izsum.it (M.S.); e.rocchegiani@izsum.it (E.R.); g.blasi@izsum.it (G.B.); 2 Alifax S.r.l., Via Francesco Petrarca, 2/1, 35020 Polverara, Padova, Italy; stefano.ceschia@alifax.com (S.C.); elisa.mitri@alifax.com (E.M.); elisa.gurian@alifax.com (E.G.)

**Keywords:** *Salmonella* spp., *Salmonella* spp. serogroups, *S*. Typhi, ATR-FTIR spectroscopy, I-dOne, identification algorithm

## Abstract

Salmonellosis is the second-most commonly reported foodborne gastrointestinal infection in the European Union and a major contributor to foodborne outbreaks globally. *Salmonella* serotyping differentiates typhoidal strains requiring antibiotic therapy (e.g., serovars Typhi, Paratyphi A, Paratyphi B-d-tartrate negative, Paratyphi C) from typically self-limiting non-typhoidal *Salmonella* (NTS) strains, making precise identification essential for appropriate treatment and epidemiological tracking. At the same time, the ability to identify the serogroup of *Salmonella*, regardless of which of the above two groups it belongs to, provides an important initial epidemiological indication that is useful for case management by competent health authorities. This study evaluates the effectiveness of ATR-FTIR spectroscopy coupled with a machine learning algorithm to identify four key *Salmonella enterica* serogroups (B, C1, D1—including typhoidal strains such as *S*. Typhi—and E1) directly from solid monomicrobial cultures without sample pretreatment. The system was paired with I-dOne software v2.2 already able to detect *Salmonella* spp., possibly leading to the characterisation of both the species and serotype from one colony. The multivariate classification model was trained and validated with 248 strains, with an overall accuracy of >98% over 113 samples. This approach offers a potential rapid alternative for clinical labs without serotyping facilities.

## 1. Introduction

*Salmonella* is a Gram-negative, facultative anaerobic bacillus with flagella and, therefore, capable of mobility [1]—except for *S*. Gallinarum, which is not motile [2]—comprising 2659 serovars [3]. Due to this variability and numerousness, *Salmonella*’s nomenclature has been debated for many years. In 2005, the Judicial Commission of the International Committee on the Systematics of Prokaryotes issued an opinion to resolve all of the discrepancies [4]. According to this, the *Salmonella* genus contains only two species: *Salmonella enterica* and *Salmonella bongori*. *Salmonella enterica* is divided into six different subspecies that present different biochemical characteristics, named (or assigned Roman numerals) as follows: enterica (I), salamae (II), arizonae (IIIa), diarizonae (IIIb), houtenae (IV), and indica (VI). *Salmonella bongori* has no subspecies [1]. *S. enterica* subsp. *enterica* and subsp. *salamae* serovars are principally related to warm-blooded animals, while the other *S. enterica* subspecies serovars, along with *S. bongori* serovars, are mainly associated with cold-blooded animals and the environment [1]. However, this distinction is not always clear, due to the ubiquity of this pathogen in the environment.

The important antigenic characteristics of *Salmonella* for serological tests are divided into three main types: the O-antigen (also called the somatic antigen), the H-antigen (also called the flagellar antigen), and the Vi-antigen (also called the capsular antigen). All *Salmonella* strains, regardless of the species or subspecies to which they belong, therefore need to be serotyped in order to identify first the serogroup (i.e., the serological group that includes *Salmonella* strains sharing the same somatic antigen (O-antigen)) and then the serovar (i.e., *Salmonella* strains sharing the same somatic antigen (O-antigen) with other strains of the same serogroup that differ from one another in their combination of flagellar antigens (first- and second-phase H-antigens)). The Vi-antigen, which confers more virulence to the strains that possess it than to those without it, may be present in only three *Salmonella* serovars: *S*. Typhi (9,12,[Vi]:d:-), *S*. Paratyphi C (6,7,[Vi]:c:1,5), and *S*. Dublin (1,9,12,[Vi]:g,p:-). Therefore, the antigenic formula of *Salmonella* spp. consists of these three types of antigens, reported in the following way: O-antigen, Vi-antigen (if present), first-phase H-antigens, and second-phase H-antigens. A total of 2659 serovars of *Salmonella* have been identified to date, distributed as *S. enterica* (2637 serovars: subsp. *enterica* (1586), subsp. *salamae* (522), subsp. *arizonae* (102), subsp. *diarizonae* (338), subsp. *houtenae* (76), subsp. *indica* (13)), and *S. bongori* (22 serovars), according to Supplement 2008–2010 (no. 48) [3] to the White–Kauffmann–Le Minor (WKL, formerly “Kauffmann–White”) scheme [1], which is the current gold-standard reference method to determine *Salmonella* serovars.

Based on the human clinical syndromes caused by *Salmonella*, it is possible to identify two groups, typhoidal *Salmonella* and non-typhoidal *Salmonella* (NTS), which require the adoption of different therapeutic approaches and different prophylactic measures for patients by a competent health authority. Strains belonging to the first group, including the serovars Typhi and Paratyphi (A, B d-tartrate negative, C), are responsible for enteric fever and are associated with a high number of fatal cases; therefore, they always need to be treated with antibiotics [5]. The latter group, composed of the remaining strains, including the d-tartrate positive Paratyphi B variant Java, and referred to as minor *Salmonella*, is associated with different clinical syndromes of variable severity, from generally self-limiting gastroenteric symptoms that generally require only supportive therapy to rarer invasive diseases such as bacteraemia, endovascular infection, focal infection [6], meningitis, and osteomyelitis, especially in young infants and in the immunocompromised (both adults and children) [7]. In addition, according to a circular from the Italian Ministry of Health [8], in cases of typhoidal *Salmonella* infection, enteric precautions must be applied until negative results are obtained from three consecutive stool cultures from faeces collected no less than 24 h apart and no less than 48 h from the suspension of any antibiotic. If even a single stool culture tests positive, the entire procedure must be repeated after one month. The infected individual should be removed from activities involving food handling, healthcare, or childcare until full clearance of the infection is confirmed.

In cases of minor *Salmonella* (NTS) infection, isolation of the infected patient must be applied until their clinical recovery (solid faeces) and/or until obtaining negative results of two consecutive stool cultures from faeces collected no less than 24 h apart and no less than 48 h after the suspension of any antimicrobial treatment. Even the measures against cohabitants and contacts of the infected patient are more stringent in the first than in the second case [8]. Therefore, it follows that the discrimination between typhoidal *Salmonella* infection and minor *Salmonella* (NST) infection is strictly required.

According to the European Union One Health 2022 Zoonoses Report [9,10], salmonellosis was the second-most commonly reported foodborne gastrointestinal infection in humans in the European Union and a major cause of foodborne outbreaks in European Union member states as well as non-member states. A total of 65,208 confirmed human salmonellosis cases were reported by 27 MSs in 2022, corresponding to an EU notification rate of 15.3 cases per 100,000 population. As in previous years, the top five acquired *Salmonella* serovars involved in human infections in the European Union were distributed as follows: *S*. Enteritidis (67.3%), *S*. Typhimurium (13.1%), monophasic variant of *S*. Typhimurium (1,4,[5],12:i:-) (4.3%), *S*. Infantis (2.3%), and *S*. Derby (0.89%). In Italy, despite the European trend, salmonellosis has always represented the most commonly reported foodborne gastrointestinal infection in humans, with 3302 reported cases in 2022 and a notification rate of 5.6 cases per 100,000 population [9,10].

It follows that clinical laboratories need to have easily usable and reliable identification systems that allow for the quickest possible diagnosis and the possibility of discriminating between typhoidal *Salmonella* infection, which always requires antibiotic therapy, and minor *Salmonella* (NST) infection, which is generally self-limiting and requires only supportive therapy (i.e., administration of oral rehydration solutions, lactic ferments, and probiotics) except in newborns under 3 months of age and in subjects with chronic degenerative diseases. Moreover, the serotyping of *Salmonella* strains plays an important role in terms of epidemiological surveillance, and rapid responses could help the competent authorities in the more immediate management of individual cases or outbreaks.

In clinical microbiology laboratories, *Salmonella* identification is generally performed using biochemical and serological tests, including automated systems such as the VITEK2 system (bioMérieux, Lyon, France) [11].

In recent years, matrix-assisted laser desorption/ionisation time-of-flight mass spectrometry (MALDI-TOF MS) has been introduced as a routine identification system due to its speed and accuracy [12]. Two common MALDI-TOF MS systems—the MALDI Biotyper^®^ (Bruker Daltonik GmbH, Bremen, Germany) and the VITEK MS system (bioMérieux, Lyon, France)—are routinely used in clinical laboratories.

According to Bastin et al. [13], the MALDI Biotyper could identify 100% of *Salmonella* isolates at the genus level; however, it failed to correctly identify typhoidal *Salmonella*, making it an unsuitable tool for the identification of *Salmonella* at the serovar level [14]. In the same way, according to Gyu Ri Kim et al. [15], VITEK MS is suitable for the identification of *Salmonella* at the genus level, with 100% sensitivity. However, additional tests, such as the VITEK2 system, are required to identify typhoidal *Salmonella* spp. (*Salmonella* Typhi and *Salmonella* Paratyphi A). Nevertheless, *Salmonella* Paratyphi B cannot be correctly identified at the serovar level by either the VITEK2 system or VITEK MS, and additional tests, such as traditional serological typing, are needed [15]. The other NTS serovars lack prompt characterisation methods beyond serology. Therefore, *Salmonella* typing always requires at least two different steps, the identification of the genus and the separate typing, leading to higher costs, expertise, and maintenance requirements and lengthening the wait for results.

Real-time PCR methods that can distinguish between the most common *Salmonella* serovars by detecting unique serovar-specific gene markers [16], along with multiple in silico tools to determine *Salmonella* serovars from whole-genome sequence data, have been developed [17,18]. However, these techniques, requiring specific expertise from the personnel [19] and incurring high costs in terms of the reagents and laboratory equipment used, are generally employed in second-level laboratories (i.e., regional or national) rather than in clinical first-level microbiology laboratories.

In recent years, vibrational spectroscopy techniques (e.g., infrared spectroscopy), coupled with chemometrics and multivariate machine learning algorithms, have become a highly promising tool for the rapid and accurate physicochemical characterisation and differentiation of microbes at several taxonomic levels [20,21,22,23,24,25].

Attenuated total reflectance Fourier transform infrared (ATR-FTIR) spectroscopy analyses the absorption of infrared light by biomolecules with microbial cells, providing insights into the sample’s chemical composition. ATR refers to the sampling technique for measuring samples (liquid, solid, gel-like, etc.) directly spread in tiny amounts on a mounted crystal with a high refractive index. The system allows for fast and easy measurements without extensive sample preparation. The instrument’s geometry is compact and does not require additional accessories, such as a microscope, as the focal point is intrinsically set. Briefly, the IR beam goes from the source to the sample through the optical path and the ATR crystal. The IR beam is then partially absorbed by the sample at the interface with the crystal, and the resulting spectrum reflects the chemical composition of the sample itself. Then, the crystal can be easily cleaned, if necessary, in preparation for the next analysis, making it ergonomic and safe for the operator.

**Figure 1:**
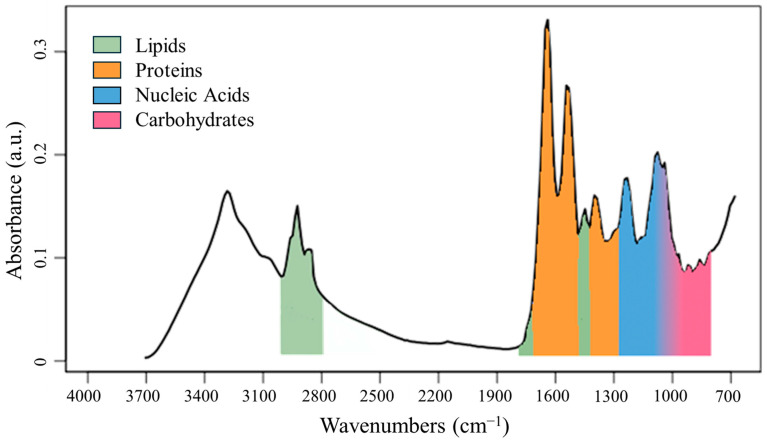
Typical ATR-FTIR spectrum of a generic microorganism and simplified band assignment.

The ATR-FTIR spectrum of an intact microorganism provides the metabolic fingerprint that reflects the macromolecular composition of cells in terms of nucleic acids, proteins, lipids, and carbohydrate levels (700–1500 cm^−1^, Figure 1) [23,26,27]. The qualitative interpretation of the spectral pattern of this region plays a significant role in the discrimination of specimens and, therefore, in the identification process.

For this purpose, I-dOne software (Alifax S.r.l., IT, Polverara, Italy) has recently been placed on the market, and it is the first CE-IVD-marked tool for the identification of 56 different microbial species or genera, including *Salmonella* spp., from colonies grown on agar media of clinical interest.

Indeed, *Salmonella enterica* strains have shown distinctive features across several serogroups and serovars under the lens of infrared spectroscopy, considering several sampling methods [24,27] and culture media [23,26,28], positioning this technique as a potential serotyping method [22]. In this regard, in addition to the actual relationship between the spectral fingerprint and the strain or the species to which it belongs, the metabolism of the isolate and its FTIR spectra can also be slightly altered by the culture media from which it originates [28]; hence, growth conditions must also be adequately considered when using FTIR for identification purposes.

The literature does not report any vibrational spectroscopic options combining species and subspecies identification. In this paper, we present the results of a feasibility study for the detection of NTS belonging to four *Salmonella enterica* serogroups (B, C1, D1, and E1) and typhoidal salmonellae (referring to *S*. Typhi from solid culture of human origin), using ATR-FTIR technology coupled with the I-dOne software v2.2, based on machine learning prediction models. This study aims to propose the potential use of this methodology in first-level clinical laboratories, which often cannot subtype *Salmonella*, as a tool to identify the prevalent serogroups in humans.

## 2. Materials and Methods

Four *Salmonella enterica* subsp. *enterica* serogroups (B, C1, D1, and E1) of major clinical interest were chosen from among the total of 47 included in the WKL scheme [1]. The study included 225 strains referable to different serovars of minor *Salmonella* (NTS), including, among others, the top five acquired *Salmonella* serovars involved in human infections in the European Union (*S*. Enteritidis, *S*. Typhimurium, monophasic variant of *S*. Typhimurium (1,4,[5],12:i:-), *S*. Infantis, and *S*. Derby). Moreover, a total of 23 strains belonging to typhoidal *Salmonella* and referable to *S*. Typhi were added (Table 1).

Only *Salmonella* strains with a complete antigenic formula, i.e., belonging to a known serogroup, were included. Overall, the database was built focusing on five main classes: the four serogroups plus the serovar *S*. Typhi (part of serogroup D1).

All of the selected *Salmonella* strains, both non-typhoidal and typhoidal, are available from the human-origin frozen culture collection of the Centro di Riferimento Regionale Patogeni Enterici, Marche region (CRRPE) of IZSUM and were obtained via the Enter-Net surveillance network, which sends *Salmonella* strains isolated from clinical samples at hospitals or private laboratories to the CRRPE for further characterisations, beginning with serotyping.

Therefore, serotyping was previously performed on the strains used in this study, in accordance with ISO/TR 6579-3:2014 [29]. *Salmonella* isolates grown as pure 18–24 h cultures in tryptic soy agar (TSA) tubes from the original culture and confirmed to be *Salmonella* through biochemical characterisation using BioMérieux strips (rapid ID 32 E, system for identification of *Enterobacteriaceae* in 4 h), comprising a series of miniaturised biochemical tests, were first tested by saline drop slide agglutination to screen for O-rough isolates (i.e., auto-agglutinating strains).

Antigenic formulae and serovars were determined using the WKL scheme by slide agglutination of the individual strain with commercially available *Salmonella* antisera (Statens Serum Institut, Copenaghen, Denmark) against the somatic (O), capsular (Vi), and flagellar (H) antigens. Where required, phase inversion was induced to determine the second phase by pouring the Sven Gard medium (previously dissolved in a boiling water bath) into a Petri dish, waiting for it to solidify, and then placing 3–4 drops of flagellar serum from the already defined phase at the centre of the dish and inoculating it with a loop of culture. After incubation at 37 °C for 24 h, the culture was tested with the flagellar sera to determine the presence of the other flagellar phase, if any.

Each selected *Salmonella* strain, as mentioned above, was cultured as a pure 18–24 h culture on five different types of agar medium from the frozen culture collection according to a random pattern in order to reduce potential systematic error. The agar plates were used as follows: tryptic soy agar (TSA, IZSUM-made production, Perugia, Italy), blood agar +5% sheep blood (BA, IZSUM-made production, Perugia, Italy), MacConkey agar (MCK, IZSUM-made production, Perugia, Italy), chromogenic agar for *Salmonella* (CROM, IZSUM-made production, Perugia, Italy), and Columbia agar +5% sheep blood (COS, BioMérieux, Marcy-l’Étoile, Francia). Among the 248 samples in the list, 42 were acquired only on TSA, MCK, and COS at the Alifax S.r.l. facility (Nimis, Italy), providing a second location in the dataset.

### 2.1. Spectrum Acquisition

**Figure 2:**
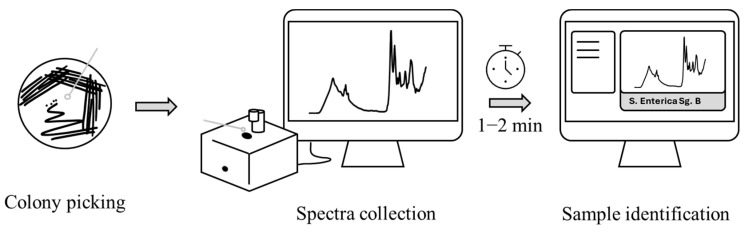
I-dOne’s workflow.

Spectra were acquired using an ATR-FTIR spectrometer (5500a Series, Agilent, Santa Clara, CA, USA) working in the mid-infrared range (4000–650 cm^−1^). The data were recorded at room temperature (25 ± 2 °C) via 64 scans at a resolution of 4 cm^−1^. Spectrum collection was carried out using I-dOne IVD software v2.2 (Alifax S.r.l., IT) to store data according to the proprietary database’s requirements using the acquisition wizard, which helps the user in the process (Figure 2). The complete procedure flows automatically and involves (i) crystal cleanliness check, (ii) background acquisition, (iii) sample deposition and spectral profile check, and (iv) spectrum acquisition. After each measurement, the ATR crystal was cleaned with a few drops of 70% *v*/*v* ethanol and wiped with tissue paper to avoid inter-sample cross-contamination. Data were collected from pure isolates on solid culture. Briefly, a small amount of sample (possibly an isolated colony) was picked with a 1 μL loop from the fourth quadrant of a culture plate and evenly spread on the ATR crystal during the sample deposition phase, followed by spectrum collection. The software itself checks the quality of the sample signal and allows for the automatic acquisition of the spectrum if it complies with the criteria defined by the manufacturer. Each acquisition takes less than two minutes. The purpose of the wizard is to guarantee standardised analytical procedures, solving the operator-dependent bias issue. For this reason, all of the acquired spectra were considered eligible for the data analysis, as they were compatible with the I-dOne software’s quality standard.

### 2.2. Dataset Construction and Metrics

The measurements were organised according to a random scheme across media and serogroups from three different operators and on three different instruments at two locations. The goal was to limit environment-, time-, instrument-, and operator-dependent biases in the dataset and the predictive algorithm, enabling better tuning of the bias–variance trade-off and generalisation of the model to unseen data [30]. Overall, about 4500 spectra were acquired, with at least 3 technical replicates for each strain in each culture medium. Raw data were reprocessed using R software (v4.0.3) [31] to build a new algorithm capable of differentiating the four *Salmonella enterica* serogroups. Preliminary exploratory data analysis for visualisation purposes involved spectral processing with the Savitzky–Golay filter to obtain the smoothed second derivative, along with vector normalisation. The algorithm pipeline was fully elaborated under patent [32], from the raw data up to the optimised model for the identification of unknown samples.

Building a multivariate predictive algorithm implies establishing and verifying a model and validating it on unknown spectra. This is generally achieved using different portions of the dataset. Table 1 reports the sample size of the databases used for training and validating the prediction model, describing the serovars included in each part. Briefly, 30 strains from each of the four serogroups and 15 from *S*. Typhi were included for the algorithm’s training, all sourced from each culture medium (135 isolates). In each class, the strains were chosen according to a proportional stratified random sampling, in order to include the greatest diversity with respect to the serovars. Where only one item was available, the distribution was random. Training the algorithm with diverse and balanced classes prevents bias towards one or more classes and allows for better model generalisation. Conversely, class weight data were used as parameters to tune the model towards *S*. Typhi versus the other cases. The remaining 113 strains served as the validation set. Since not all of the validation strains were analysed from all of the culture media, the sample size was unbalanced across this factor: 113 strains on COS, MCK, and TSA, including 71 that were also measured on both BA and CROM. It should be noted that no strains from the training set were included in the validation set, preventing overfitting and ensuring unbiased performance results.

A 10-fold cross-validation stratified sampling scheme was applied to train and test the algorithm in order to estimate the model parameters that would maximise the identification performances on the test sets. Moreover, according to the I-dOne standard workflow, if the first identification of an unknown sample is not reliable, i.e., the spectrum is dissimilar to the database, a second or even a third independent acquisition might be required. Results were given after the comparison between up to three acquisitions. In the event of low agreement, the sample was deemed to be unidentifiable. The rationale behind the acceptance criteria is covered by the patent [32]. The number of spectra per result was a performance metric that mirrors the effort required to achieve the final identification. Validation results were then obtained by running the optimised model on the validation set and following this workflow.

To benchmark the model’s performance on the validation dataset, sensitivity and accuracy percentages were retrieved for each class from the multiclass confusion matrix [33]. In a multiclass classification problem, the confusion matrix is a table that is used to evaluate the performance of a classification model by showing the numbers of true positives, false positives, false negatives, and true negatives for each class. To demonstrate the algorithm’s efficiency across the different culture media, each strain–medium combination—represented by each plate—was treated as an individual sample, simulating the results attainable through a routine evaluation method. The class-wise sensitivity counted the number of correctly identified strains versus the overall number of samples for each class. The accuracy was computed as the ratio of the correctly identified samples to the total number of strains. In a multiclass scenario with unbalanced classes, individual class sensitivity—here, the serogroups—avoided the sample size bias. This ensured that the performance of the majority classes did not overshadow the minority ones. Additionally, presenting the actual number of correctly identified isolates, stratified by culture medium, highlighted potential issues related to specific conditions. Eventual misclassifications can be commented on by considering this factor.

### 2.3. Data Clustering Visualisation

A prior step in data classification is the visualisation of relationships among spectra. Several multivariate clustering techniques allow for the definition of whether similar spectra naturally cluster with respect to classes (serogroups). When dealing with a complex dataset containing hundreds of variables per spectrum, distilling it into a clear, visually interpretable 2- or 3-dimensional plot is challenging. In addition, t-SNE (t-distributed stochastic neighbour embedding) is a nonlinear, unsupervised dimensionality reduction technique that is used specifically for visualising complex datasets in lower dimensions (in this case, 2D, referred to as features) [34]; it maps data based on similarity or underlying implicit structure in the dataset and identifies patterns, often revealing the natural tendency of data to form clusters based on shared features. This method can capture more complex, nonlinear patterns that linear methods, such as the principal component analysis, might miss. Briefly, each point represents a spectrum, and the t-SNE algorithm computes their coordinates so that very similar objects in the original space are spatially closed within this new space, and vice versa. The quality of the t-SNE clustering can be visually inspected, as the low-dimensional projection of the high-dimensional data aligns with the classes’ distinction.

## 3. Results

**Figure 3:**
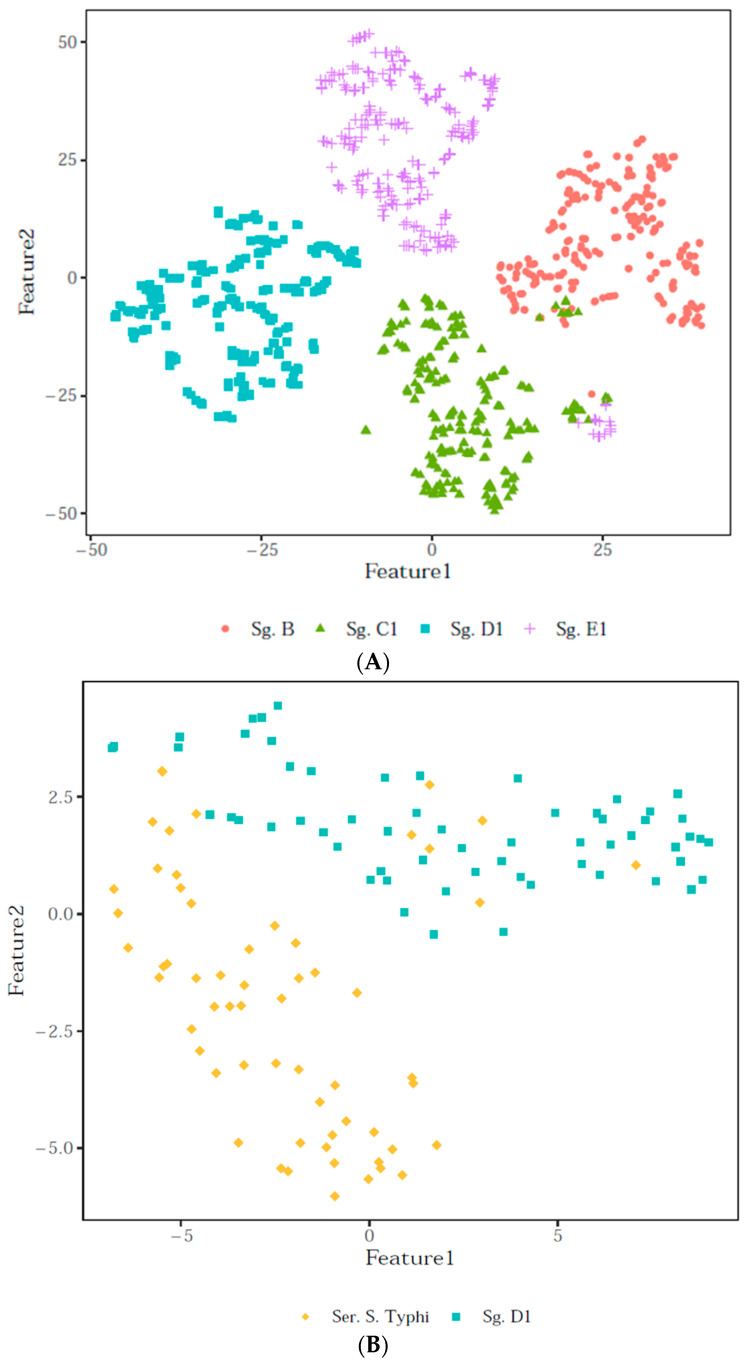
The t-SNE plots computed (**A**) on a representative subset of the database and (**B**) only for serogroup D1 and serovar *S*. Typhi, coloured by serogroup membership.

The graphical separation of serogroups was determined by the average behaviour of the pre-processed spectra, as described in Section 2. Figure 3 reports data from all of the samples and uses media as the t-SNE plots. In this case, the t-SNE chart showcased the spectral separation of the classes included in the present database. The plot colours were added according to class membership once the map had been computed in order to highlight intraclass similarity and interclass distinction. It follows that some classes (serogroups) were separable from the rest of the dataset, e.g., serogroup D1, although inter-group similarities between individual strains were possible (Figure 3A). Within serogroup D1, *S*. Typhi showed slight superimpositions with other serovars, allegedly due to their similar composition in terms of membrane decoration [35] (Figure 3B).

**Figure 4:**
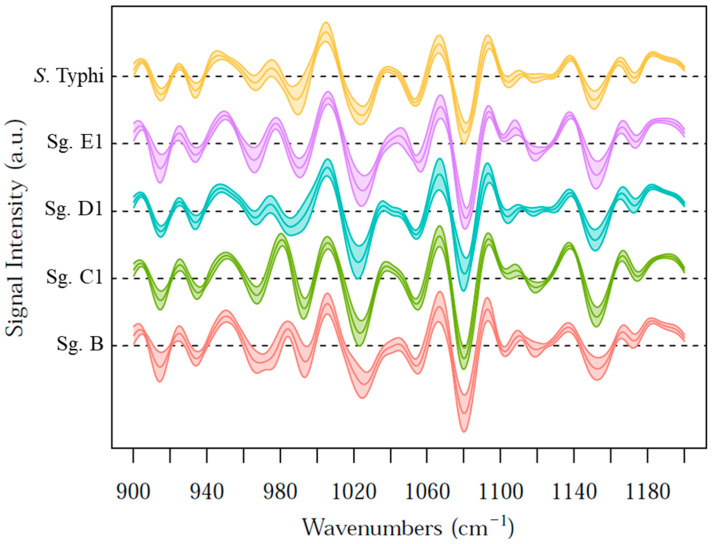
The second derivative of the fingerprint region. Data from all of the spectra and media are stacked and aggregated by class (mean and first standard deviation).

Looking at the actual pattern of the ATR-FTIR spectra, Figure 4 shows their second derivative in the fingerprint region, stacked by class membership (shadowed: mean and first standard deviation). IR spectral data are often analysed in terms of the second derivative in order to emphasise the separation of overlapping peaks, enhancing subtle intraclass variability. The average profiles show several differences across the whole *x*-axis and should be considered as fingerprints of each class. Major differences have been found in the 900–1200 cm^−1^ range, attributed to phosphate stretching vibrations, phospholipids, and carbohydrates [36].

The final performances reported in Table 2 were computed on the validation dataset, stratified by class and culture medium, whereas Table 3 reports the confusion matrix obtained for the overall dataset, irrespective of the growth conditions (see Supplementary Materials Table S1, for the complete list of results). The values express the number of strains multiplied by the culture media from which they have been analysed. A combined reading of the tables highlights the actual distribution of misclassifications. Only four strains were wrongly identified across one or more media. Serogroup C1 suffered across almost all of the media for only two strains, belonging to serovars Infantis and Choleraesuis. The other similar serovars did not show any misinterpretation; hence, this may have been due to peculiarities in their phenotype making them similar to the other serogroups. Meanwhile, mutual misclassification among serogroup D1 (*S*. Enteritidis) and serovar *S*. Typhi occurred one time each, on COS and MCK, respectively. The outputs for the same strains on the other culture media were correct. On the other hand, no strains from serogroups B and E1 were wrongly classified in this validation set. In terms of system throughput performance, all validation strains achieved confirmed identification with a single spectrum across various conditions, leading to a 2 min test per sample. Multiple acquisitions, required by I-dOne when results are unreliable, were not required for any additional measurements in these cases.

## 4. Discussion

FTIR spectroscopy is known to be capable of subtyping *Salmonella* at different taxonomic levels [37,38] and even at the phagotype level [39]. In this context, FTIR is counted among the state-of-the-art methods from a phenotypic point of view [40] while also finding indirect relationships from a genotypic point of view [37,41] or at the clonal level [22]. For instance, Novais et al. [42] described the potential use of FTIR for *Klebsiella pneumoniae* typing, which is useful for monitoring outbreaks and supporting the control of nosocomial infections. Baldauf [37,43], Preisner [38], and Cordovana [40,44] successfully developed algorithms for the identification of several *Salmonella enterica* serogroups or serovars using transmission or attenuated total reflectance FTIR spectroscopy, discussing the role played by culture media in the spectral profile and the need to expand the database according to the expected use and the experimental framework. However, these cases always require sample pretreatment or time-consuming spectrum collection, as well as proposing offline identification algorithms, which are available only after complete data collection.

The present study aimed at evaluating the capability of ATR-FTIR spectroscopy coupled with I-dOne (Alifax S.r.l.) software to subtype among four *Salmonella* serogroups (B, C1, D1, and E1) and the serovar Typhi. The choice of serogroups included in this study was based on clinical incidence [9,45] and public health impact [46].

Figure 3 and Figure 4 highlight how ATR-FTIR can effectively distinguish among serogroups, focusing on the 900–1200 cm^−1^ range, reflecting the structural variations in the polysaccharide portion [47,48]. This spectral region can correspond to the O-antigen’s chemical structure, in which the lipopolysaccharide chains underpin the specificity of each serogroup, allowing for class attribution, as in the reference typing methods. Bacterial lipopolysaccharides consist of a lipid portion, responsible for the toxicity of the germ (called Lipid A), and a polysaccharide portion, responsible for the antigenic specificity of the soma (comprising the core and O-antigen). Lipid A is hidden within the membrane and has a similar structure across different Gram-negative bacterial species. Conversely, the polysaccharide portion is located on the outer membrane of Gram-negative bacteria, including *Salmonella*. The diversity and uniqueness of each O-antigen characterising a specific *Salmonella* serogroup depends on this external polysaccharide portion included in the O-antigen, along with its peculiar sugar sequence [35,49]. Several studies suggest that structural variations in the O-antigen are among the primary factors enabling FTIR spectroscopy to distinguish bacterial strains at the infra-species level and for other genera. For instance, Beutin was able to discriminate *Escherichia coli* O4 from O123 via the O-antigen signature in the FTIR spectra [50], Kuhm highlighted the role of the polysaccharide region in differentiating *Yersinia enterocolitica* subtypes [51], and Vogt found high concordance between genetic clustering and the 900–1200 cm^−1^ plus 700–900 cm^−1^ FTIR regions for typing the *E. cloacae complex* strains for real-time surveillance and outbreak analysis [48].

The identification algorithm was built with balanced classes to ensure that every class was adequately represented. An exception was made for *S*. Typhi, due to the rarity of the strains. A balanced database allows the performance of the model to be fairly evaluated in all classes. Otherwise, following the natural epidemiology of the *Salmonella* serogroups would result in a severely unbalanced situation, leading to a bias towards the minority class in favour of the most represented one.

The sample size distribution, favouring the training set over the validation set, allowed intra- and interclass variability to be included in the algorithm as best as possible. The larger the training database, the greater the possibility of recognising new and unknown strains. It must be said, however, that misclassifications cannot be excluded a priori, no matter what phenotypic identification method is considered.

An overall robust sensitivity was obtained over different culture media, with the class sensitivity always higher than 97%, and an overall accuracy of 98.3%. Since only four strains were misclassified, and errors were spread over one or more culture media, no specific strain- or media-driven bias was detected. Possibly, the mistakes may have been due to peculiar features that the individual strains displayed at the infrared level; deeper characterisation shall be carried out on these specific cases. Indeed, no diagnostic test for microbial identification is perfect. Although there are many sensitive and specific tests available for identifying microorganisms, it is important to recognise that no test can ensure 100% accuracy, not even more established techniques such as MALDI-TOF, which is still unable to distinguish *E. coli* from *Shigella* due to their intrinsic similarity [52]. Moreover, the Statens Serum Institut (SSI) sera, commonly used for *Salmonella* serotyping and widely regarded as a reliable reference for typing different serovars according to the Kauffmann–White scheme, do not offer 100% sensitivity and specificity; in fact, they can successfully type 99% of the known *Salmonella* serovars, as described on the SSI Diagnostica website.

We chose to use two blood-based agar media (BA, IZSUM-made production; COS, BioMérieux) that, in principle, should not differ in composition; however, depending on the manufacturer, they may differ in terms of the proportions in the recipe or in the use of different raw materials. Including a commercial substrate in the design allowed us to monitor eventual drifts in the identification due to the media not included in I-dOne’s use specifications. Indeed, the fact that differences in identification outcomes for the same strain were especially found when comparing these two-agar media is likely explainable based on these—albeit slight—differences in terms of recipe, which may induce metabolic profiles and ATR-FTIR spectra that can overlap with the IR signatures of IR-related classes. This issue is strain specific. As the ATR-FTIR spectrum is a combined reflection of both phenotypes and growth media, predicting the spectral profile of a brand-new sample is challenging. Amiali et al. [41] emphasised that, for a robust classification algorithm, a database should account for all key variables: strain origin, culture medium, environmental conditions, and instrumental drift, among others. All of these possible foreseen factors should be introduced to the framework in a non-confounded fashion. Only by expanding the training dataset over time with diverse strains and media is it possible to fully capture the statistical behaviour of each class, improving the algorithm’s discriminative capacity. In this study, class variance was expanded using serovars of diverse phenotypes, along with several culture media. The choice of the culture media was suitable to design an algorithm primarily intended for clinical microbiology routines, where standard screening culture media can be used, without the need for a medium-specific identification workflow.

This study also aimed to distinguish between NTS and typhoidal *Salmonella* strains, given the different therapeutic approaches that these two types of infection require and the different prophylactic measures that must be adopted for patients by the competent health authority [8].

We started considering *S*. Typhi due to its higher incidence in Italy than the Paratyphi serovars (A, d-tartrate-negative B, C) (in the period 2016–2021: 214 strains of *S*. Typhi, 47 of *S*. Paratyphi A, 22 of *S*. Paratyphi B, and 18 of *S*. Paratyphi C), along with its multidrug resistance (MDR) capability, higher than that of paratyphoidal serovars (29.7% vs. 12.0%) [45].

Although the sample size of *S*. Typhi was unbalanced with respect to other cases, the results were all consistent, except for one case predicted as a member of serogroup D1—formerly correct—in MCK medium. One similar misclassification occurred for another D1 strain flagged as *S*. Typhi in COS medium. These errors, although severe, can be justified considering that *S*. Typhi belongs to serogroup D1 and high physicochemical similarities at the FTIR level are plausible; hence, to date, overlapping between the spectral regions of the two classes cannot be ruled out. In this sense, misclassifications could be linked not to systematic errors with respect to growth conditions but to the peculiar behaviour of the strains in the media. Moreover, in this case, it is important to recall the interaction between the isolates and the growth media, as the latter inevitably alters the metabolism of the former. Despite efforts to include the greatest possible cross-factor variability, outliers cannot be excluded a priori.

In the absence of additional *S*. Typhi strains, the algorithm could be retrained and tested using alternative oversampling techniques or synthetic data in order to balance the overall dataset across classes and to refine the algorithm’s performance. Although this can be a common choice in machine learning methods, it is not straightforward to guess biological variability in synthetic data, and reusing the same spectra multiple times does not introduce realistic or beneficial variability. For this reason, such a route was not chosen. Actual clinical strains would always represent a proper reference in terms of training and validation results; hence, misclassification might be overcome by retraining the algorithm over an enriched database with more strains and growth conditions.

In addition, it is worth mentioning the possibility of further refining the identification of the serovar Paratyphi B variant Java from among the other strains of serogroup B. This serovar displays spectral features similar to those of serogroup B, as mentioned by Cordovana et al. [40], and preliminary results from the current database indicate a sensitivity of 80% in the discrimination of the Paratyphi B variant Java versus the other serovars of group B. However, the spectral overlapping between Paratyphi B and other serovars of serogroup B would lead to misclassifications from both sides due to the high degree of similarity between the spectra of these classes. This is explained by the similarity in terms of capsular chemistry within serogroup B, which prevents a clear distinction, possibly related to the diversity within *S*. Paratyphi B clones and the high genetic heterogeneity within this serovar, making it similar to other serovars [40,53,54,55]. Accordingly, it is premature to include the Paratyphi B variant Java in a potential list of reliably identifiable serogroups. On the other hand, whereas the d-tartrate-positive Paratyphi B variant Java is NTS, the possibility of ruling out Paratyphi B d-tartrate-negative (which is among the typhoidal salmonellae) could help clinicians with patient management [56,57]. Therefore, further database enrichment should be carried out in order to better define the spectral features of this class and improve its identification performance.

The additional purpose of this study was to design an algorithm that could be implemented in an all-in-one system that streamlines the full process from the data acquisition to the analysis and simply returns the result of the identification, as the I-dOne CE-IVD software v2.2 does. The inclusion of the developed algorithm within or consecutively to I-dOne’s workflow has several major potential benefits. On the one hand, it makes it possible to take advantage of the automated quality control of the spectral profile prior to any acquisition; on the other, there is the possibility of combining strain identification at the spp. level and subsequent typing with the same instrument and the same pipeline within minutes: a spectroscopy-based solution not yet proposed in the state of the art.

In addition to the implementation of the *Salmonella* identification route, the ATR-FTIR technique coupled with I-dOne software merits additional comments. It ensures rapid sample processing without the need for pretreatment, reagents, or concerns about carryover, as the I-dOne software automatically verifies the crystal’s cleanliness before and after each measurement. As ATR-FTIR analyses one sample at a time, the progress of each measurement can be monitored in real time. The software’s built-in wizard enables the operator to visually track the spectrum’s development immediately after sample deposition and to eventually adjust to ensure the sample’s homogeneity on the ATR crystal, meeting I-dOne’s stringent standards. With the present algorithm, only one spectrum for each strain was enough to return a reliable identification. Considering that it takes less than 2 min for a complete identification, this system qualifies as quick and easy to use, speeding up the performance.

## 5. Conclusions

For *Salmonella* spp., serotyping analysis through the WKL scheme still represents the current gold-standard reference method; however, since it is expensive and time-consuming and requires considerable expertise and visual interpretation by the operator in the interpretation of the results, it is rightly restricted only to regional or national reference laboratories.

Nevertheless, preliminary discrimination at the serogroup level, performed by routine clinical laboratories through rapid and user-friendly methods, represents the first important indication for epidemiological investigations and for the control of foodborne outbreaks, as well as for the clinical management of salmonellosis.

In this study, we found that the ATR-FTIR system could represent a reliable and promising method for the discrimination of *Salmonella* spp. at the serogroup level.

The advantages of this system’s use are related both to the user-friendly nature of the automated software—which provides fast results in minutes directly from the pure culture on agar plates, avoiding any operator-dependent bias—and to the equipment, which requires almost no maintenance and does not require reagents.

For these reasons, the ATR-FTIR methodology seems to be easily implementable within routine laboratory activities as an alternative and rapid method for initial *Salmonella* typing at the serogroup level. The I-dOne suite offers the possibility to combine species and subspecies identification within the same workflow and with the same instrument, streamlining the process. Moreover, proper data collection could result in a flexible method for use in various laboratory setups.

Further studies on improving the discrimination of *S*. Typhi from other serovars of serogroup D1, as well as the identifiability of paratyphoid (*S*. Paratyphi A, *S*. Paratyphi B d-tartrate-negative, and *S*. Paratyphi C) and other serogroups, will be needed.

Limitations remain with regard to the refined identification of other serovars, since every machine learning-based method requires a large and well-described database of isolates, which could be overcome in the future. At the same time, growth conditions (e.g., incubation setup, culture medium) must be considered and included in the study design in order to identify the boundary conditions for the application of the method. This study highlights how expanding the cross-factors towards several setups is feasible, making the ATR-FTIR approach scalable.

Concurrently, further studies should be conducted on how the antibiotic resistance profiles of the *Salmonella* strains could influence the ability of the ATR-FTIR method to identify them.

## 6. Patents

The I-dOne workflow and data analysis pipeline are covered by patent [32]; hence, the details cannot be revealed.

## Figures and Tables

**Table 1:** List of the strains included in this study, grouped by serogroup and detailed by serovar and sample size for model training and validation.

Serogroup	Serovar	N (248)	N Train (135)	N Validation (113)
B	Agona	3	2	1
	Brandenburg	6	1	5
	Bredeney	5	3	2
	Chester	1	1	0
	Coeln	3	2	1
	Derby	6	3	3
	Essen	1	0	1
	MVST ^1^	30	7	23
	Paratyphi B variant Java	22	7	15
	Saintpaul	1	1	0
	Stanleyville	1	0	1
	Typhimurium	9	1	8
	Typhimurium O:5-	2	2	0
C1	Birkenhead	1	1	0
	Braenderup	1	1	0
	Choleraesuis	3	2	1
	Choleraesuis variant Kunzendorf	1	0	1
	Colorado	1	1	0
	Infantis	19	12	7
	Isangi	1	0	1
	Jerusalem	1	1	0
	Kenya	1	1	0
	Livingstone	2	2	0
	Mbandaka	2	2	0
	Mikawasima	1	0	1
	Montevideo	1	0	1
	Oritamerin	1	0	1
	Rissen	5	3	2
	Strathcona	6	4	2
D1	Enteritidis	19	14	5
	Israel	1	0	1
	Kapemba	6	3	3
	Napoli	12	10	2
	Panama	4	3	1
	Typhi	23	15	8
E1	Anatum	11	6	5
	Give	9	7	2
	London	20	14	6
	Muenster	4	2	2
	Orion	2	1	1

^1^ MVST = monophasic variant of *Salmonella* Typhimurium.

**Table 2:** Sensitivity and accuracy performances stratified by the class and culture medium on the validation dataset. For clarity, performances were computed on the totals (row- and column-wise) and not on the individual class–medium combination. Culture media grouped as BA (blood agar), MCK (MacConkey agar), TSA (tryptic soy agar), CROM (chromogenic agar), and COS (Columbia agar + 5% sheep blood). The number of correct identifications is reported versus the total number of cases (in brackets).

Class	No.	Sensitivity	BA	MCK	TSA	CROM	COS
Sg. B	248 (248)	100.0%	34 (34)	60 (60)	60 (60)	34 (34)	60 (60)
Sg. C1	65 (71)	91.5%	10 (10)	15 (17)	16 (17)	9 (10)	15 (17)
Sg. D1	55 (56)	98.2%	10 (10)	12 (12)	12 (12)	10 (10)	11 (12)
Sg. E1	68 (68)	100.0%	10 (10)	16 (16)	16 (16)	10 (10)	16 (16)
Ser. Typhi	37 (38)	97.4%	7 (7)	7 (8)	8 (8)	7 (7)	8 (8)
Total	473 (481)		71 (71)	110 (113)	112 (113)	70 (71)	110 (113)
Accuracy	98.3%		100.0%	97.3%	99.1%	98.6%	97.3%

**Table 3:** Confusion matrix for the whole dataset. Values express the number of strains multiplied by the culture media from which they have been analysed. Columns show the actual reference class, while rows show the predictions.

			Reference ID			
		Sg. B	Sg. C1	Sg. D1	Sg. E1	Ser. Typhi
Prediction	Sg. B	248	5	0	0	0
	Sg. C1	0	65	0	0	0
	Sg. D1	0	0	55	0	1
	Sg. E1	0	1	0	68	0
	Ser. Typhi	0	0	1	0	37
	Total	248	71	56	68	38

## Data Availability

The original contributions presented in the study are included in the article/Supplementary Materials, further inquiries can be directed to the corresponding author.

## Supplementary Materials

The following supporting information can be downloaded at https://www.mdpi.com/article/10.3390/microorganisms12112318/s1: Table S1. Identification results for the 113 samples included in the validation set.

## References

[1] Grimont P.A.D., Weill F.-X. (2007). Antigenic Formulae of the Salmonella Serovars.

[2] Wales A., Lawes J. (2023). JMM Profile: Salmonella Enterica Serovar Gallinarum, Biovars Pullorum and Gallinarum: This Article Is Part of the JMM Profiles Collection. J. Med. Microbiol..

[3] Issenhuth-Jeanjean S., Roggentin P., Mikoleit M., Guibourdenche M., De Pinna E., Nair S., Fields P.I., Weill F.-X. (2014). Supplement 2008–2010 (No. 48) to the White–Kauffmann–Le Minor Scheme. Res. Microbiol..

[4] Tindall B.J., Grimont P.A.D., Garrity G.M., Euzéby J.P. (2005). Nomenclature and Taxonomy of the Genus Salmonella. Int. J. Syst. Evol. Microbiol..

[5] Wang X., Biswas S., Paudyal N., Pan H., Li X., Fang W., Yue M. (2019). Antibiotic Resistance in Salmonella Typhimurium Isolates Recovered From the Food Chain Through National Antimicrobial Resistance Monitoring System Between 1996 and 2016. Front. Microbiol..

[6] Sánchez-Vargas F.M., Abu-El-Haija M.A., Gómez-Duarte O.G. (2011). Salmonella Infections: An Update on Epidemiology, Management, and Prevention. Travel Med. Infect. Dis..

[7] Wen S.C., Best E., Nourse C. (2017). Non-typhoidal *Salmonella* Infections in Children: Review of Literature and Recommendations for Management. J. Paediatr. Child Health.

[8] Protocollo 400.3/26/1189. Ministero della Salute della Repubblica Italiana. Misure di Profilassi per Esigenze di Sanita’ Pubblica Provvedimenti da Adottare nei Confronti di Soggetti Affetti da Alcune Malattie Infettive e Nei Confronti di Loro Conviventi o Contatti. https://www.salute.gov.it/portale/malattieInfettive/archivioNormativaMalattieInfettive.jsp?lingua=italiano&anno=1998&btnCerca=cerca.

[9] European Food Safety Authority, European Centre for Disease Prevention and Control (2023). European Centre for Disease Prevention and Control (ECDC) The European Union One Health 2022 Zoonoses Report. EFSA J..

[10] Survellaince Atlas of Infectious Diseases. https://atlas.ecdc.europa.eu/public/index.aspx.

[11] Deng J., Fu L., Wang R., Yu N., Ding X., Jiang L., Fang Y., Jiang C., Lin L., Wang Y. (2014). Comparison of MALDI-TOF MS, Gene Sequencing and the Vitek 2 for Identification of Seventy-Three Clinical Isolates of Enteropathogens. J. Thorac. Dis..

[12] Tsuchida S., Nakayama T. (2022). MALDI-Based Mass Spectrometry in Clinical Testing: Focus on Bacterial Identification. Appl. Sci..

[13] Bastin B., Bird P., Benzinger M.J., Crowley E., Agin J., Goins D., Sohier D., Timke M., Shi G., Kostrzewa M. (2019). Confirmation and Identification of *Salmonella* spp., *Cronobacter* spp., and Other Gram-Negative Organisms by the Bruker MALDI Biotyper Method: Collaborative Study Method Extension to Include Campylobacter Species, Revised First Action 2017.09. J. AOAC Int..

[14] Kuhns M., Zautner A.E., Rabsch W., Zimmermann O., Weig M., Bader O., Groß U. (2012). Rapid Discrimination of Salmonella Enterica Serovar Typhi from Other Serovars by MALDI-TOF Mass Spectrometry. PLoS ONE.

[15] Kim G.R., Kim S.H., Kim E.-Y., Park E.H., Hwang I.Y., Jeong S.H., Kim H.S., Kim Y.A., Uh Y., Shin K.S. (2022). Performance of MALDI-TOF Mass Spectrometry (VITEK MS) in the Identification of Salmonella Species. Microorganisms.

[16] Yang S.-M., Kim E., Kim D., Kim H.-B., Baek J., Ko S., Kim D., Yoon H., Kim H.-Y. (2021). Rapid Real-Time Polymerase Chain Reaction for Salmonella Serotyping Based on Novel Unique Gene Markers by Pangenome Analysis. Front. Microbiol..

[17] Zhang S., Den Bakker H.C., Li S., Chen J., Dinsmore B.A., Lane C., Lauer A.C., Fields P.I., Deng X. (2019). SeqSero2: Rapid and Improved *Salmonella* Serotype Determination Using Whole-Genome Sequencing Data. Appl. Environ. Microbiol..

[18] Yoshida C.E., Kruczkiewicz P., Laing C.R., Lingohr E.J., Gannon V.P.J., Nash J.H.E., Taboada E.N. (2016). The Salmonella In Silico Typing Resource (SISTR): An Open Web-Accessible Tool for Rapidly Typing and Subtyping Draft Salmonella Genome Assemblies. PLoS ONE.

[19] Mellmann A., Andersen P.S., Bletz S., Friedrich A.W., Kohl T.A., Lilje B., Niemann S., Prior K., Rossen J.W., Harmsen D. (2017). High Interlaboratory Reproducibility and Accuracy of Next-Generation-Sequencing-Based Bacterial Genotyping in a Ring Trial. J. Clin. Microbiol..

[20] Classification and Identification of Bacteria by Fourier-Transform Infrared Spectroscopy|Microbiology Society. https://www.microbiologyresearch.org/content/journal/micro/10.1099/00221287-137-1-69.

[21] Horbach I., Naumann D., Fehrenbach F.J. (1988). Simultaneous Infections with Different Serogroups of Legionella Pneumophila Investigated by Routine Methods and Fourier Transform Infrared Spectroscopy. J. Clin. Microbiol..

[22] Mariey L., Signolle J.P., Amiel C., Travert J. (2001). Discrimination, Classification, Identification of Microorganisms Using FTIR Spectroscopy and Chemometrics. Vib. Spectrosc..

[23] Naumann D., Helm D., Labischinski H. (1991). Microbiological Characterizations by FT-IR Spectroscopy. Nature.

[24] Zarnowiec P., Lechowicz L., Czerwonka G., Kaca W. (2015). Fourier Transform Infrared Spectroscopy (FTIR) as a Tool for the Identification and Differentiation of Pathogenic Bacteria. Curr. Med. Chem..

[25] Van Belkum A., Tassios P.T., Dijkshoorn L., Haeggman S., Cookson B., Fry N.K., Fussing V., Green J., Feil E., Gerner-Smidt P. (2007). Guidelines for the Validation and Application of Typing Methods for Use in Bacterial Epidemiology. Clin. Microbiol. Infect..

[26] Quintelas C., Ferreira E.C., Lopes J.A., Sousa C. (2018). An Overview of the Evolution of Infrared Spectroscopy Applied to Bacterial Typing. Biotechnol. J..

[27] Vallieres E., Quach C., Lam L., Rallu F., Langella M., Sedman J., Raymond M., Lebel P., Ismail A. (2017). Attenuated Total Reflectance Fourier Transform Infrared Spectroscopy for Rapid Identification of Non-Fermenting Gram-Negative Bacilli Isolated from Patients with Cystic Fibrosis. Open Forum Infect. Dis..

[28] Amiali N.M., Golding G.R., Sedman J., Simor A.E., Ismail A.A. (2011). Rapid Identification of Community-Associated Methicillin-Resistant Staphylococcus Aureus by Fourier Transform Infrared Spectroscopy. Diagn. Microbiol. Infect. Dis..

[29] (2014). Microbiology of the Food Chain—Horizontal Method for the Detection, Enumeration and Serotyping of Salmonella—Part 3: Guidelines for Serotyping of *Salmonella* spp.

[30] Brereton R.G., Jansen J., Lopes J., Marini F., Pomerantsev A., Rodionova O., Roger J.M., Walczak B., Tauler R. (2017). Chemometrics in Analytical Chemistry—Part I: History, Experimental Design and Data Analysis Tools. Anal. Bioanal. Chem..

[31] R Core Team (2021). R: A Language and Environment for Statistical Computing.

[32] Galiano P. (2019). Method and System to Identify Microorganisms.

[33] Beleites C., Neugebauer U., Bocklitz T., Krafft C., Popp J. (2013). Sample Size Planning for Classification Models. Anal. Chim. Acta.

[34] Van der Maaten L., Hinton G. (2008). Visualizing Data Using T-SNE. J. Mach. Learn. Res..

[35] Liu B., Knirel Y.A., Feng L., Perepelov A.V., Senchenkova S.N., Reeves P.R., Wang L. (2014). Structural Diversity in *Salmonella* O Antigens and Its Genetic Basis. FEMS Microbiol. Rev..

[36] Parikh S.J., Chorover J. (2008). ATR-FTIR Study of Lipopolysaccharides at Mineral Surfaces. Colloids Surf. B Biointerfaces.

[37] Baldauf N., Rodriguez Romo L., Yousef A., Rodriguez-Saona L. (2006). Differentiation of Selected Salmonella Enterica Serovars by Fourier Transform Mid-Infrared Spectroscopy. Appl. Spectrosc..

[38] Preisner O.E., Menezes J.C., Guiomar R., Machado J., Lopes J.A. (2012). Discrimination of Salmonella Enterica Serotypes by Fourier Transform Infrared Spectroscopy. Food Res. Int..

[39] Kim S., Kim H., Reuhs B.L., Mauer L.J. (2006). Differentiation of Outer Membrane Proteins from Salmonellaenterica Serotypes Using Fourier Transform Infrared Spectroscopy and Chemometrics. Lett. Appl. Microbiol..

[40] Cordovana M., Mauder N., Join-Lambert O., Gravey F., LeHello S., Auzou M., Pitti M., Zoppi S., Buhl M., Steinmann J. (2022). Machine Learning-Based Typing of Salmonella Enterica O-Serogroups by the Fourier-Transform Infrared (FTIR) Spectroscopy-Based IR Biotyper System. J. Microbiol. Methods.

[41] Amiali N.M., Mulvey M.R., Sedman J., Simor A.E., Ismail A.A. (2007). Epidemiological Typing of Methicillin-Resistant *Staphylococcus Aureus* Strains by Fourier Transform Infrared Spectroscopy. J. Microbiol. Methods.

[42] Novais Â., Gonçalves A.B., Ribeiro T.G., Freitas A.R., Méndez G., Mancera L., Read A., Alves V., López-Cerero L., Rodríguez-Baño J. (2024). Development and Validation of a Quick, Automated, and Reproducible ATR FT-IR Spectroscopy Machine-Learning Model for Klebsiella Pneumoniae Typing. J. Clin. Microbiol..

[43] Baldauf N.A., Rodriguez-Romo L.A., Männig A., Yousef A.E., Rodriguez-Saona L.E. (2007). Effect of Selective Growth Media on the Differentiation of Salmonella Enterica Serovars by Fourier-Transform Mid-Infrared Spectroscopy. J. Microbiol. Methods.

[44] Cordovana M., Mauder N., Kostrzewa M., Wille A., Rojak S., Hagen R.M., Ambretti S., Pongolini S., Soliani L., Justesen U.S. (2021). Classification of Salmonella Enterica of the (Para-)Typhoid Fever Group by Fourier-Transform Infrared (FTIR) Spectroscopy. Microorganisms.

[45] Lucarelli C., García-Fernández A., Dionisi A., Owczarek S., Arena S., Fortini D., Errico G., Maraglino F., Pilati S., Palamara A. (2024). Sorveglianza Nazionale delle Infezioni da Salmonella, Campylobacter, Shigella e Yersinia. Dati Enter-Net Italia 2016–2021. (Rapporti ISS Sorveglianza RIS-1/2024).

[46] Hagedoorn N.N., Murthy S., Birkhold M., Marchello C.S., Crump J.A. (2024). The Vacc-iNTS Consortium Collaborators Prevalence and Distribution of Non-Typhoidal *Salmonella enterica* Serogroups and Serovars Isolated from Normally Sterile Sites: A Global Systematic Review. Epidemiol. Infect..

[47] Sukprasert J., Thumanu K., Phung-on I., Jirarungsatean C., Erickson L.E., Tuitemwong P., Tuitemwong K. (2020). Synchrotron FTIR Light Reveals Signal Changes of Biofunctionalized Magnetic Nanoparticle Attachment on *Salmonella* sp. J. Nanomater..

[48] Vogt S., Löffler K., Dinkelacker A.G., Bader B., Autenrieth I.B., Peter S., Liese J. (2019). Fourier-Transform Infrared (FTIR) Spectroscopy for Typing of Clinical Enterobacter Cloacae Complex Isolates. Front. Microbiol..

[49] Graziani C., Galetta P., Busani L., Dionisi A.M., Filetici E., Ricci A., Caprioli A., Luzzi I. Infezioni Da Salmonella: Diagnostica, Epidemiologia e Sorveglianza. 2005, Rapporti ISTISAN 05/27, 49p. https://www.researchgate.net/publication/282608632_Le_infezioni_da_Salmonella_diagnostica_epidemiologia_e_sorveglianza-Salmonella_infections_diagnosis_epidemiology_and_surveillance.

[50] Beutin L., Wang Q., Naumann D., Han W., Krause G., Leomil L., Wang L., Feng L. (2007). Relationship between O-Antigen Subtypes, Bacterial Surface Structures and O-Antigen Gene Clusters in Escherichia Coli O123 Strains Carrying Genes for Shiga Toxins and Intimin. J. Med. Microbiol..

[51] Kuhm A.E., Suter D., Felleisen R., Rau J. (2009). Identification of *Yersinia enterocolitica* at the Species and Subspecies Levels by Fourier Transform Infrared Spectroscopy. Appl. Environ. Microbiol..

[52] Rychert J. (2019). Benefits and Limitations of MALDI-TOF Mass Spectrometry for the Identification of Microorganisms. Clin. Mass. Spectrom..

[53] Selander R.K., Beltran P., Smith N.H., Barker R.M., Crichton P.B., Old D.C., Musser J.M., Whittam T.S. (1990). Genetic Population Structure, Clonal Phylogeny, and Pathogenicity of Salmonella Paratyphi B. Infect. Immun..

[54] Barker R.M., Kearney G.M., Nicholson P., Blair A.L., Porter R.C., Crichton P.B. (1988). Types of Salmonella Paratyphi B and Their Phylogenetic Significance. J. Med. Microbiol..

[55] Achtman M., Wain J., Weill F.-X., Nair S., Zhou Z., Sangal V., Krauland M.G., Hale J.L., Harbottle H., Uesbeck A. (2012). Multilocus Sequence Typing as a Replacement for Serotyping in Salmonella Enterica. PLoS Pathog..

[56] Malorny B., Bunge C., Helmuth R. (2003). Discrimination of D-Tartrate-Fermenting and -Nonfermenting *Salmonella enterica* subsp. *enterica* Isolates by Genotypic and Phenotypic Methods. J. Clin. Microbiol..

[57] Connor T.R., Owen S.V., Langridge G., Connell S., Nair S., Reuter S., Dallman T.J., Corander J., Tabing K.C., Le Hello S. (2016). What’s in a Name? Species-Wide Whole-Genome Sequencing Resolves Invasive and Noninvasive Lineages of *Salmonella enterica* Serotype Paratyphi B. mBio.

