# Supplementary material for: Identification of Salmonella Serogroups and Distinction Between Typhoidal and Non-Typhoidal Salmonella Based on ATR-FTIR Spectroscopy
Source: Microorganisms. 2024 Nov 14;12(11):2318. doi: 10.3390/microorganisms12112318 (PMC11596249; doi:10.3390/microorganisms12112318)
Supplement: Supplementary file 1 [file microorganisms-12-02318-s001.zip › microorganisms-3304252-supplementary.pdf]

Supplementary Materials

Identification of *Salmonella* Serogroups and Distinction Between Typhoidal and Non-Typhoidal *Salmonella* Based on ATR-FTIR Spectroscopy

Maira Napoleoni [1,*,†], Stefano Ceschia [2], Elisa Mitri [2], Elisa Eleonora Beneitez [1], Valentina Silenzi [1], Monica Staffolani [1], Elena Rocchegiani [1], Giuliana Blasi [1] and Elisa Gurian [2,†]

[1] Centro di Riferimento Regionale Patogeni Enterici Marche, Istituto Zooprofilattico Sperimentale dell'Umbria e delle Marche "Togo Rosati", Via Maestri del Lavoro, 7, 62029 Tolentino, Macerata, Italy; e.beneitez@izsum.it (E.E.B.); v.silenzi@izsum.it (V.S.); m.staffolani@izsum.it (M.S.); e.rocchegiani@izsum.it (E.R.); g.blasi@izsum.it (G.B.)
[2] Alifax S.r.l., Via Francesco Petrarca, 2/1, 35020 Polverara, Padova, Italy; stefano.ceschia@alifax.com (S.C.); elisa.mitri@alifax.com (E.M.); elisa.gurian@alifax.com (E.G.)
* Correspondence: m.napoleoni@izsum.it; Tel.: +39-07332-62206
† These authors contributed equally to this work.

Table S1. Identification results for the 113 samples included in the validation set.

#	Internal ID	Medium	Serovar ID	Reference ID	Prediction
**1**	Sample_1	COS	Derby	B	B
**2**	Sample_1	MCK	Derby	B	B
**3**	Sample_1	TSA	Derby	B	B
**4**	Sample_2	BA	London	E1	E1
**5**	Sample_2	COS	London	E1	E1
**6**	Sample_2	CROM	London	E1	E1
**7**	Sample_2	MCK	London	E1	E1
**8**	Sample_2	TSA	London	E1	E1
**9**	Sample_3	BA	Paratyphi B var. Java	B	B
**10**	Sample_3	COS	Paratyphi B var. Java	B	B
**11**	Sample_3	CROM	Paratyphi B var. Java	B	B
**12**	Sample_3	MCK	Paratyphi B var. Java	B	B
**13**	Sample_3	TSA	Paratyphi B var. Java	B	B
**14**	Sample_4	BA	Rissen	C1	C1
**15**	Sample_4	COS	Rissen	C1	C1
**16**	Sample_4	CROM	Rissen	C1	C1
**17**	Sample_4	MCK	Rissen	C1	C1
**18**	Sample_4	TSA	Rissen	C1	C1
**19**	Sample_5	COS	Typhimurium	B	B
**20**	Sample_5	MCK	Typhimurium	B	B
**21**	Sample_5	TSA	Typhimurium	B	B
**22**	Sample_6	BA	Brandenburg	B	B
**23**	Sample_6	COS	Brandenburg	B	B

#	Internal ID	Medium	Serovar ID	Reference ID	Prediction
**24**	Sample_6	CROM	Brandenburg	B	B
**25**	Sample_6	MCK	Brandenburg	B	B
**26**	Sample_6	TSA	Brandenburg	B	B
**27**	Sample_7	BA	Enteritidis	D1	D1
**28**	Sample_7	COS	Enteritidis	D1	Typhi
**29**	Sample_7	CROM	Enteritidis	D1	D1
**30**	Sample_7	MCK	Enteritidis	D1	D1
**31**	Sample_7	TSA	Enteritidis	D1	D1
**32**	Sample_8	BA	Enteritidis	D1	D1
**33**	Sample_8	COS	Enteritidis	D1	D1
**34**	Sample_8	CROM	Enteritidis	D1	D1
**35**	Sample_8	MCK	Enteritidis	D1	D1
**36**	Sample_8	TSA	Enteritidis	D1	D1
**37**	Sample_9	BA	Enteritidis	D1	D1
**38**	Sample_9	COS	Enteritidis	D1	D1
**39**	Sample_9	CROM	Enteritidis	D1	D1
**40**	Sample_9	MCK	Enteritidis	D1	D1
**41**	Sample_9	TSA	Enteritidis	D1	D1
**42**	Sample_10	BA	Typhi	Typhi	Typhi
**43**	Sample_10	COS	Typhi	Typhi	Typhi
**44**	Sample_10	CROM	Typhi	Typhi	Typhi
**45**	Sample_10	MCK	Typhi	Typhi	Typhi
**46**	Sample_10	TSA	Typhi	Typhi	Typhi
**47**	Sample_11	BA	Infantis	C1	C1
**48**	Sample_11	COS	Infantis	C1	C1
**49**	Sample_11	CROM	Infantis	C1	C1
**50**	Sample_11	MCK	Infantis	C1	C1
**51**	Sample_11	TSA	Infantis	C1	C1
**52**	Sample_12	BA	VMST	B	B
**53**	Sample_12	COS	VMST	B	B
**54**	Sample_12	CROM	VMST	B	B
**55**	Sample_12	MCK	VMST	B	B
**56**	Sample_12	TSA	VMST	B	B
**57**	Sample_13	BA	Typhi	Typhi	Typhi
**58**	Sample_13	COS	Typhi	Typhi	Typhi
**59**	Sample_13	CROM	Typhi	Typhi	Typhi
**60**	Sample_13	MCK	Typhi	Typhi	Typhi
**61**	Sample_13	TSA	Typhi	Typhi	Typhi
**62**	Sample_14	BA	Brandenburg	B	B
**63**	Sample_14	COS	Brandenburg	B	B

#	Internal ID	Medium	Serovar ID	Reference ID	Prediction
**64**	Sample_14	CROM	Brandenburg	B	B
**65**	Sample_14	MCK	Brandenburg	B	B
**66**	Sample_14	TSA	Brandenburg	B	B
**67**	Sample_15	BA	Panama	D1	D1
**68**	Sample_15	COS	Panama	D1	D1
**69**	Sample_15	CROM	Panama	D1	D1
**70**	Sample_15	MCK	Panama	D1	D1
**71**	Sample_15	TSA	Panama	D1	D1
**72**	Sample_16	BA	Derby	B	B
**73**	Sample_16	COS	Derby	B	B
**74**	Sample_16	CROM	Derby	B	B
**75**	Sample_16	MCK	Derby	B	B
**76**	Sample_16	TSA	Derby	B	B
**77**	Sample_17	BA	Infantis	C1	C1
**78**	Sample_17	COS	Infantis	C1	C1
**79**	Sample_17	CROM	Infantis	C1	C1
**80**	Sample_17	MCK	Infantis	C1	C1
**81**	Sample_17	TSA	Infantis	C1	C1
**82**	Sample_18	BA	Infantis	C1	C1
**83**	Sample_18	COS	Infantis	C1	C1
**84**	Sample_18	CROM	Infantis	C1	C1
**85**	Sample_18	MCK	Infantis	C1	C1
**86**	Sample_18	TSA	Infantis	C1	C1
**87**	Sample_19	BA	VMST	B	B
**88**	Sample_19	COS	VMST	B	B
**89**	Sample_19	CROM	VMST	B	B
**90**	Sample_19	MCK	VMST	B	B
**91**	Sample_19	TSA	VMST	B	B
**92**	Sample_20	BA	Typhi	Typhi	Typhi
**93**	Sample_20	COS	Typhi	Typhi	Typhi
**94**	Sample_20	CROM	Typhi	Typhi	Typhi
**95**	Sample_20	MCK	Typhi	Typhi	Typhi
**96**	Sample_20	TSA	Typhi	Typhi	Typhi
**97**	Sample_21	BA	Typhimurium	B	B
**98**	Sample_21	COS	Typhimurium	B	B
**99**	Sample_21	CROM	Typhimurium	B	B
**100**	Sample_21	MCK	Typhimurium	B	B
**101**	Sample_21	TSA	Typhimurium	B	B
**102**	Sample_22	BA	Typhimurium	B	B
**103**	Sample_22	COS	Typhimurium	B	B

#	Internal ID	Medium	Serovar ID	Reference ID	Prediction
**104**	Sample_22	CROM	Typhimurium	B	B
**105**	Sample_22	MCK	Typhimurium	B	B
**106**	Sample_22	TSA	Typhimurium	B	B
**107**	Sample_23	BA	Enteritidis	D1	D1
**108**	Sample_23	COS	Enteritidis	D1	D1
**109**	Sample_23	CROM	Enteritidis	D1	D1
**110**	Sample_23	MCK	Enteritidis	D1	D1
**111**	Sample_23	TSA	Enteritidis	D1	D1
**112**	Sample_24	BA	Brandenburg	B	B
**113**	Sample_24	COS	Brandenburg	B	B
**114**	Sample_24	CROM	Brandenburg	B	B
**115**	Sample_24	MCK	Brandenburg	B	B
**116**	Sample_24	TSA	Brandenburg	B	B
**117**	Sample_25	BA	Montevideo	C1	C1
**118**	Sample_25	COS	Montevideo	C1	C1
**119**	Sample_25	CROM	Montevideo	C1	C1
**120**	Sample_25	MCK	Montevideo	C1	C1
**121**	Sample_25	TSA	Montevideo	C1	C1
**122**	Sample_26	BA	London	E1	E1
**123**	Sample_26	COS	London	E1	E1
**124**	Sample_26	CROM	London	E1	E1
**125**	Sample_26	MCK	London	E1	E1
**126**	Sample_26	TSA	London	E1	E1
**127**	Sample_27	BA	Isangi	C1	C1
**128**	Sample_27	COS	Isangi	C1	C1
**129**	Sample_27	CROM	Isangi	C1	C1
**130**	Sample_27	MCK	Isangi	C1	C1
**131**	Sample_27	TSA	Isangi	C1	C1
**132**	Sample_28	BA	Paratyphi B var. Java	B	B
**133**	Sample_28	COS	Paratyphi B var. Java	B	B
**134**	Sample_28	CROM	Paratyphi B var. Java	B	B
**135**	Sample_28	MCK	Paratyphi B var. Java	B	B
**136**	Sample_28	TSA	Paratyphi B var. Java	B	B
**137**	Sample_29	BA	Brandenburg	B	B
**138**	Sample_29	COS	Brandenburg	B	B
**139**	Sample_29	CROM	Brandenburg	B	B
**140**	Sample_29	MCK	Brandenburg	B	B
**141**	Sample_29	TSA	Brandenburg	B	B
**142**	Sample_30	BA	Essen	B	B
**143**	Sample_30	COS	Essen	B	B

#	Internal ID	Medium	Serovar ID	Reference ID	Prediction
**144**	Sample_30	CROM	Essen	B	B
**145**	Sample_30	MCK	Essen	B	B
**146**	Sample_30	TSA	Essen	B	B
**147**	Sample_31	BA	Typhimurium	B	B
**148**	Sample_31	COS	Typhimurium	B	B
**149**	Sample_31	CROM	Typhimurium	B	B
**150**	Sample_31	MCK	Typhimurium	B	B
**151**	Sample_31	TSA	Typhimurium	B	B
**152**	Sample_32	BA	Infantis	C1	C1
**153**	Sample_32	COS	Infantis	C1	B
**154**	Sample_32	CROM	Infantis	C1	E1
**155**	Sample_32	MCK	Infantis	C1	B
**156**	Sample_32	TSA	Infantis	C1	C1
**157**	Sample_33	BA	Typhi	Typhi	Typhi
**158**	Sample_33	COS	Typhi	Typhi	Typhi
**159**	Sample_33	CROM	Typhi	Typhi	Typhi
**160**	Sample_33	MCK	Typhi	Typhi	Typhi
**161**	Sample_33	TSA	Typhi	Typhi	Typhi
**162**	Sample_34	BA	Napoli	D1	D1
**163**	Sample_34	COS	Napoli	D1	D1
**164**	Sample_34	CROM	Napoli	D1	D1
**165**	Sample_34	MCK	Napoli	D1	D1
**166**	Sample_34	TSA	Napoli	D1	D1
**167**	Sample_35	BA	Paratyphi B var. Java	B	B
**168**	Sample_35	COS	Paratyphi B var. Java	B	B
**169**	Sample_35	CROM	Paratyphi B var. Java	B	B
**170**	Sample_35	MCK	Paratyphi B var. Java	B	B
**171**	Sample_35	TSA	Paratyphi B var. Java	B	B
**172**	Sample_36	BA	Mikawasima	C1	C1
**173**	Sample_36	COS	Mikawasima	C1	C1
**174**	Sample_36	CROM	Mikawasima	C1	C1
**175**	Sample_36	MCK	Mikawasima	C1	C1
**176**	Sample_36	TSA	Mikawasima	C1	C1
**177**	Sample_37	BA	Bredeney	B	B
**178**	Sample_37	COS	Bredeney	B	B
**179**	Sample_37	CROM	Bredeney	B	B
**180**	Sample_37	MCK	Bredeney	B	B
**181**	Sample_37	TSA	Bredeney	B	B
**182**	Sample_38	BA	London	E1	E1
**183**	Sample_38	COS	London	E1	E1

#	Internal ID	Medium	Serovar ID	Reference ID	Prediction
**184**	Sample_38	CROM	London	E1	E1
**185**	Sample_38	MCK	London	E1	E1
**186**	Sample_38	TSA	London	E1	E1
**187**	Sample_39	BA	Anatum	E1	E1
**188**	Sample_39	COS	Anatum	E1	E1
**189**	Sample_39	CROM	Anatum	E1	E1
**190**	Sample_39	MCK	Anatum	E1	E1
**191**	Sample_39	TSA	Anatum	E1	E1
**192**	Sample_40	BA	Anatum	E1	E1
**193**	Sample_40	COS	Anatum	E1	E1
**194**	Sample_40	CROM	Anatum	E1	E1
**195**	Sample_40	MCK	Anatum	E1	E1
**196**	Sample_40	TSA	Anatum	E1	E1
**197**	Sample_41	BA	Brandenburg	B	B
**198**	Sample_41	COS	Brandenburg	B	B
**199**	Sample_41	CROM	Brandenburg	B	B
**200**	Sample_41	MCK	Brandenburg	B	B
**201**	Sample_41	TSA	Brandenburg	B	B
**202**	Sample_42	BA	Paratyphi B var. Java	B	B
**203**	Sample_42	COS	Paratyphi B var. Java	B	B
**204**	Sample_42	CROM	Paratyphi B var. Java	B	B
**205**	Sample_42	MCK	Paratyphi B var. Java	B	B
**206**	Sample_42	TSA	Paratyphi B var. Java	B	B
**207**	Sample_43	BA	Napoli	D1	D1
**208**	Sample_43	COS	Napoli	D1	D1
**209**	Sample_43	CROM	Napoli	D1	D1
**210**	Sample_43	MCK	Napoli	D1	D1
**211**	Sample_43	TSA	Napoli	D1	D1
**212**	Sample_44	BA	Typhi	Typhi	Typhi
**213**	Sample_44	COS	Typhi	Typhi	Typhi
**214**	Sample_44	CROM	Typhi	Typhi	Typhi
**215**	Sample_44	MCK	Typhi	Typhi	Typhi
**216**	Sample_44	TSA	Typhi	Typhi	Typhi
**217**	Sample_45	BA	Paratyphi B var. Java	B	B
**218**	Sample_45	COS	Paratyphi B var. Java	B	B
**219**	Sample_45	CROM	Paratyphi B var. Java	B	B
**220**	Sample_45	MCK	Paratyphi B var. Java	B	B
**221**	Sample_45	TSA	Paratyphi B var. Java	B	B
**222**	Sample_46	BA	Strathcona	C1	C1
**223**	Sample_46	COS	Strathcona	C1	C1

#	Internal ID	Medium	Serovar ID	Reference ID	Prediction
**224**	Sample_46	CROM	Strathcona	C1	C1
**225**	Sample_46	MCK	Strathcona	C1	C1
**226**	Sample_46	TSA	Strathcona	C1	C1
**227**	Sample_47	BA	Paratyphi B var. Java	B	B
**228**	Sample_47	COS	Paratyphi B var. Java	B	B
**229**	Sample_47	CROM	Paratyphi B var. Java	B	B
**230**	Sample_47	MCK	Paratyphi B var. Java	B	B
**231**	Sample_47	TSA	Paratyphi B var. Java	B	B
**232**	Sample_48	BA	Muenster	E1	E1
**233**	Sample_48	COS	Muenster	E1	E1
**234**	Sample_48	CROM	Muenster	E1	E1
**235**	Sample_48	MCK	Muenster	E1	E1
**236**	Sample_48	TSA	Muenster	E1	E1
**237**	Sample_49	BA	Paratyphi B var. Java	B	B
**238**	Sample_49	COS	Paratyphi B var. Java	B	B
**239**	Sample_49	CROM	Paratyphi B var. Java	B	B
**240**	Sample_49	MCK	Paratyphi B var. Java	B	B
**241**	Sample_49	TSA	Paratyphi B var. Java	B	B
**242**	Sample_50	BA	Paratyphi B var. Java	B	B
**243**	Sample_50	COS	Paratyphi B var. Java	B	B
**244**	Sample_50	CROM	Paratyphi B var. Java	B	B
**245**	Sample_50	MCK	Paratyphi B var. Java	B	B
**246**	Sample_50	TSA	Paratyphi B var. Java	B	B
**247**	Sample_51	BA	Agona	B	B
**248**	Sample_51	COS	Agona	B	B
**249**	Sample_51	CROM	Agona	B	B
**250**	Sample_51	MCK	Agona	B	B
**251**	Sample_51	TSA	Agona	B	B
**252**	Sample_52	BA	Bredeney	B	B
**253**	Sample_52	COS	Bredeney	B	B
**254**	Sample_52	CROM	Bredeney	B	B
**255**	Sample_52	MCK	Bredeney	B	B
**256**	Sample_52	TSA	Bredeney	B	B
**257**	Sample_53	BA	Kapemba	D1	D1
**258**	Sample_53	COS	Kapemba	D1	D1
**259**	Sample_53	CROM	Kapemba	D1	D1
**260**	Sample_53	MCK	Kapemba	D1	D1
**261**	Sample_53	TSA	Kapemba	D1	D1
**262**	Sample_54	BA	London	E1	E1
**263**	Sample_54	COS	London	E1	E1

#	Internal ID	Medium	Serovar ID	Reference ID	Prediction
**264**	Sample_54	CROM	London	E1	E1
**265**	Sample_54	MCK	London	E1	E1
**266**	Sample_54	TSA	London	E1	E1
**267**	Sample_55	BA	VMST	B	B
**268**	Sample_55	COS	VMST	B	B
**269**	Sample_55	CROM	VMST	B	B
**270**	Sample_55	MCK	VMST	B	B
**271**	Sample_55	TSA	VMST	B	B
**272**	Sample_56	BA	Give	E1	E1
**273**	Sample_56	COS	Give	E1	E1
**274**	Sample_56	CROM	Give	E1	E1
**275**	Sample_56	MCK	Give	E1	E1
**276**	Sample_56	TSA	Give	E1	E1
**277**	Sample_57	BA	Typhi	Typhi	Typhi
**278**	Sample_57	COS	Typhi	Typhi	Typhi
**279**	Sample_57	CROM	Typhi	Typhi	Typhi
**280**	Sample_57	MCK	Typhi	Typhi	Typhi
**281**	Sample_57	TSA	Typhi	Typhi	Typhi
**282**	Sample_58	BA	Orion	E1	E1
**283**	Sample_58	COS	Orion	E1	E1
**284**	Sample_58	CROM	Orion	E1	E1
**285**	Sample_58	MCK	Orion	E1	E1
**286**	Sample_58	TSA	Orion	E1	E1
**287**	Sample_59	BA	Paratyphi B var. Java	B	B
**288**	Sample_59	COS	Paratyphi B var. Java	B	B
**289**	Sample_59	CROM	Paratyphi B var. Java	B	B
**290**	Sample_59	MCK	Paratyphi B var. Java	B	B
**291**	Sample_59	TSA	Paratyphi B var. Java	B	B
**292**	Sample_60	BA	Enteritidis	D1	D1
**293**	Sample_60	COS	Enteritidis	D1	D1
**294**	Sample_60	CROM	Enteritidis	D1	D1
**295**	Sample_60	MCK	Enteritidis	D1	D1
**296**	Sample_60	TSA	Enteritidis	D1	D1
**297**	Sample_61	BA	Paratyphi B var. Java	B	B
**298**	Sample_61	COS	Paratyphi B var. Java	B	B
**299**	Sample_61	CROM	Paratyphi B var. Java	B	B
**300**	Sample_61	MCK	Paratyphi B var. Java	B	B
**301**	Sample_61	TSA	Paratyphi B var. Java	B	B
**302**	Sample_62	BA	Typhi	Typhi	Typhi
**303**	Sample_62	COS	Typhi	Typhi	Typhi

#	Internal ID	Medium	Serovar ID	Reference ID	Prediction
**304**	Sample_62	CROM	Typhi	Typhi	Typhi
**305**	Sample_62	MCK	Typhi	Typhi	Typhi
**306**	Sample_62	TSA	Typhi	Typhi	Typhi
**307**	Sample_63	BA	Coeln	B	B
**308**	Sample_63	COS	Coeln	B	B
**309**	Sample_63	CROM	Coeln	B	B
**310**	Sample_63	MCK	Coeln	B	B
**311**	Sample_63	TSA	Coeln	B	B
**312**	Sample_64	BA	Give	E1	E1
**313**	Sample_64	COS	Give	E1	E1
**314**	Sample_64	CROM	Give	E1	E1
**315**	Sample_64	MCK	Give	E1	E1
**316**	Sample_64	TSA	Give	E1	E1
**317**	Sample_65	BA	Kapemba	D1	D1
**318**	Sample_65	COS	Kapemba	D1	D1
**319**	Sample_65	CROM	Kapemba	D1	D1
**320**	Sample_65	MCK	Kapemba	D1	D1
**321**	Sample_65	TSA	Kapemba	D1	D1
**322**	Sample_66	BA	VMST	B	B
**323**	Sample_66	COS	VMST	B	B
**324**	Sample_66	CROM	VMST	B	B
**325**	Sample_66	MCK	VMST	B	B
**326**	Sample_66	TSA	VMST	B	B
**327**	Sample_67	BA	Infantis	C1	C1
**328**	Sample_67	COS	Infantis	C1	C1
**329**	Sample_67	CROM	Infantis	C1	C1
**330**	Sample_67	MCK	Infantis	C1	C1
**331**	Sample_67	TSA	Infantis	C1	C1
**332**	Sample_68	BA	VMST	B	B
**333**	Sample_68	COS	VMST	B	B
**334**	Sample_68	CROM	VMST	B	B
**335**	Sample_68	MCK	VMST	B	B
**336**	Sample_68	TSA	VMST	B	B
**337**	Sample_69	BA	VMST	B	B
**338**	Sample_69	COS	VMST	B	B
**339**	Sample_69	CROM	VMST	B	B
**340**	Sample_69	MCK	VMST	B	B
**341**	Sample_69	TSA	VMST	B	B
**342**	Sample_70	BA	Paratyphi B var. Java	B	B
**343**	Sample_70	COS	Paratyphi B var. Java	B	B

#	Internal ID	Medium	Serovar ID	Reference ID	Prediction
**344**	Sample_70	CROM	Paratyphi B var. Java	B	B
**345**	Sample_70	MCK	Paratyphi B var. Java	B	B
**346**	Sample_70	TSA	Paratyphi B var. Java	B	B
**347**	Sample_71	COS	Typhimurium	B	B
**348**	Sample_71	MCK	Typhimurium	B	B
**349**	Sample_71	TSA	Typhimurium	B	B
**350**	Sample_72	COS	VMST	B	B
**351**	Sample_72	MCK	VMST	B	B
**352**	Sample_72	TSA	VMST	B	B
**353**	Sample_73	COS	Infantis	C1	C1
**354**	Sample_73	MCK	Infantis	C1	C1
**355**	Sample_73	TSA	Infantis	C1	C1
**356**	Sample_74	COS	Choleraesuis	C1	C1
**357**	Sample_74	MCK	Choleraesuis	C1	C1
**358**	Sample_74	TSA	Choleraesuis	C1	C1
**359**	Sample_75	COS	Typhi	Typhi	Typhi
**360**	Sample_75	MCK	Typhi	Typhi	D1
**361**	Sample_75	TSA	Typhi	Typhi	Typhi
**362**	Sample_76	COS	Kapemba	D1	D1
**363**	Sample_76	MCK	Kapemba	D1	D1
**364**	Sample_76	TSA	Kapemba	D1	D1
**365**	Sample_77	COS	Muenster	E1	E1
**366**	Sample_77	MCK	Muenster	E1	E1
**367**	Sample_77	TSA	Muenster	E1	E1
**368**	Sample_78	COS	Anatum	E1	E1
**369**	Sample_78	MCK	Anatum	E1	E1
**370**	Sample_78	TSA	Anatum	E1	E1
**371**	Sample_79	BA	Paratyphi B var. Java	B	B
**372**	Sample_79	COS	Paratyphi B var. Java	B	B
**373**	Sample_79	CROM	Paratyphi B var. Java	B	B
**374**	Sample_79	MCK	Paratyphi B var. Java	B	B
**375**	Sample_79	TSA	Paratyphi B var. Java	B	B
**376**	Sample_80	BA	Paratyphi B var. Java	B	B
**377**	Sample_80	COS	Paratyphi B var. Java	B	B
**378**	Sample_80	CROM	Paratyphi B var. Java	B	B
**379**	Sample_80	MCK	Paratyphi B var. Java	B	B
**380**	Sample_80	TSA	Paratyphi B var. Java	B	B
**381**	Sample_81	BA	Paratyphi B var. Java	B	B
**382**	Sample_81	COS	Paratyphi B var. Java	B	B
**383**	Sample_81	CROM	Paratyphi B var. Java	B	B

#	Internal ID	Medium	Serovar ID	Reference ID	Prediction
**384**	Sample_81	MCK	Paratyphi B var. Java	B	B
**385**	Sample_81	TSA	Paratyphi B var. Java	B	B
**386**	Sample_82	COS	VMST	B	B
**387**	Sample_82	MCK	VMST	B	B
**388**	Sample_82	TSA	VMST	B	B
**389**	Sample_83	COS	VMST	B	B
**390**	Sample_83	MCK	VMST	B	B
**391**	Sample_83	TSA	VMST	B	B
**392**	Sample_84	COS	VMST	B	B
**393**	Sample_84	MCK	VMST	B	B
**394**	Sample_84	TSA	VMST	B	B
**395**	Sample_85	COS	Derby	B	B
**396**	Sample_85	MCK	Derby	B	B
**397**	Sample_85	TSA	Derby	B	B
**398**	Sample_86	COS	Stanleyville	B	B
**399**	Sample_86	MCK	Stanleyville	B	B
**400**	Sample_86	TSA	Stanleyville	B	B
**401**	Sample_87	COS	Infantis	C1	C1
**402**	Sample_87	MCK	Infantis	C1	C1
**403**	Sample_87	TSA	Infantis	C1	C1
**404**	Sample_88	COS	Typhimurium	B	B
**405**	Sample_88	MCK	Typhimurium	B	B
**406**	Sample_88	TSA	Typhimurium	B	B
**407**	Sample_89	COS	London	E1	E1
**408**	Sample_89	MCK	London	E1	E1
**409**	Sample_89	TSA	London	E1	E1
**410**	Sample_90	COS	VMST	B	B
**411**	Sample_90	MCK	VMST	B	B
**412**	Sample_90	TSA	VMST	B	B
**413**	Sample_91	COS	Paratyphi B var. Java	B	B
**414**	Sample_91	MCK	Paratyphi B var. Java	B	B
**415**	Sample_91	TSA	Paratyphi B var. Java	B	B
**416**	Sample_92	COS	VMST	B	B
**417**	Sample_92	MCK	VMST	B	B
**418**	Sample_92	TSA	VMST	B	B
**419**	Sample_93	COS	VMST	B	B
**420**	Sample_93	MCK	VMST	B	B
**421**	Sample_93	TSA	VMST	B	B
**422**	Sample_94	COS	VMST	B	B
**423**	Sample_94	MCK	VMST	B	B

#	Internal ID	Medium	Serovar ID	Reference ID	Prediction
**424**	Sample_94	TSA	VMST	B	B
**425**	Sample_95	COS	VMST	B	B
**426**	Sample_95	MCK	VMST	B	B
**427**	Sample_95	TSA	VMST	B	B
**428**	Sample_96	COS	Oritamerin	C1	C1
**429**	Sample_96	MCK	Oritamerin	C1	C1
**430**	Sample_96	TSA	Oritamerin	C1	C1
**431**	Sample_97	COS	VMST	B	B
**432**	Sample_97	MCK	VMST	B	B
**433**	Sample_97	TSA	VMST	B	B
**434**	Sample_98	COS	VMST	B	B
**435**	Sample_98	MCK	VMST	B	B
**436**	Sample_98	TSA	VMST	B	B
**437**	Sample_99	COS	VMST	B	B
**438**	Sample_99	MCK	VMST	B	B
**439**	Sample_99	TSA	VMST	B	B
**440**	Sample_100	COS	VMST	B	B
**441**	Sample_100	MCK	VMST	B	B
**442**	Sample_100	TSA	VMST	B	B
**443**	Sample_101	COS	Choleraesius	C1	B
**444**	Sample_101	MCK	Choleraesius	C1	B
**445**	Sample_101	TSA	Choleraesius	C1	B
**446**	Sample_102	COS	VMST	B	B
**447**	Sample_102	MCK	VMST	B	B
**448**	Sample_102	TSA	VMST	B	B
**449**	Sample_103	COS	VMST	B	B
**450**	Sample_103	MCK	VMST	B	B
**451**	Sample_103	TSA	VMST	B	B
**452**	Sample_104	COS	Typhimurium	B	B
**453**	Sample_104	MCK	Typhimurium	B	B
**454**	Sample_104	TSA	Typhimurium	B	B
**455**	Sample_105	COS	VMST	B	B
**456**	Sample_105	MCK	VMST	B	B
**457**	Sample_105	TSA	VMST	B	B
**458**	Sample_106	COS	Israel	D1	D1
**459**	Sample_106	MCK	Israel	D1	D1
**460**	Sample_106	TSA	Israel	D1	D1
**461**	Sample_107	COS	Strathcona	C1	C1
**462**	Sample_107	MCK	Strathcona	C1	C1
**463**	Sample_107	TSA	Strathcona	C1	C1

#	Internal ID	Medium	Serovar ID	Reference ID	Prediction
**464**	Sample_108	COS	Rissen	C1	C1
**465**	Sample_108	MCK	Rissen	C1	C1
**466**	Sample_108	TSA	Rissen	C1	C1
**467**	Sample_109	COS	London	E1	E1
**468**	Sample_109	MCK	London	E1	E1
**469**	Sample_109	TSA	London	E1	E1
**470**	Sample_110	COS	VMST	B	B
**471**	Sample_110	MCK	VMST	B	B
**472**	Sample_110	TSA	VMST	B	B
**473**	Sample_111	COS	Typhimurium	B	B
**474**	Sample_111	MCK	Typhimurium	B	B
**475**	Sample_111	TSA	Typhimurium	B	B
**476**	Sample_112	COS	Anatum	E1	E1
**477**	Sample_112	MCK	Anatum	E1	E1
**478**	Sample_112	TSA	Anatum	E1	E1
**479**	Sample_113	COS	Anatum	E1	E1
**480**	Sample_113	MCK	Anatum	E1	E1
**481**	Sample_113	TSA	Anatum	E1	E1
